# Antidiabetic activity of aqueous root extract of *Ichnocarpus frutescens* in streptozotocin-nicotinamide induced type-II diabetes in rats

**DOI:** 10.4103/0253-7613.40484

**Published:** 2008

**Authors:** Rakesh Barik, Sanjay Jain, Deep Qwatra, Amit Joshi, Girraj Sharan Tripathi, Ravi Goyal

**Affiliations:** Department of Pharmacognosy, Smriti College of Pharmaceutical Education, 4/1, Pipliya Kakkad, Mayakhedi Road, Nipania, Indore - 452 010, MP, India

**Keywords:** Antidiabetic oral glucose tolerance test, *Ichnocarpus frutescens*, streptozotocin-nicotinamide induced type-II diabetes mellitus

## Abstract

**Objective:**

To evaluate the antidiabetic activity of aqueous extract of roots of *Ichnocarpus frutescens* in streptozotocin-nicotinamide induced type-II diabetes in rats.

**Materials and Methods:**

Streptozotocin-nicotinamide induced type-II diabetic rats (*n* = 6) were administered aqueous root extract (250 and 500 mg/kg, p.o.) of *Ichnocarpus frutescens* or vehicle (gum acacia solution) or standard drug glibenclamide (0.25 mg/kg) for 15 days. Blood samples were collected by retro-orbital puncture and were analyzed for serum glucose on days 0, 5, 10, and 15 by using glucose oxidase-peroxidase reactive strips and a glucometer. For oral glucose tolerance test, glucose (2 g/kg, p.o.) was administered to nondiabetic control rats and the rats treated with glibenclamide (10 mg/kg, p.o.) and aqueous root extract of *Ichnocarpus frutescens*. The serum glucose levels were analyzed at 0, 30, 60, and 120 min after drug administration. The effect of the extract on the body weight of the diabetic rats was also observed.

**Results:**

The aqueous root extract of *Ichnocarpus frutescens* (250 and 500 mg/kg, p.o.) induced significant reduction (*P* < 0.05) of fasting blood glucose levels in streptozotocin-nicotinamide induced type-II diabetic rats on the 10^th^ and 15^th^ days. In the oral glucose tolerance test, the extract increased the glucose tolerance. It also brought about an increase in the body weight of diabetic rats.

**Conclusion:**

It is concluded that *Ichnocarpus frutescens* has significant antidiabetic activity as it lowers the fasting blood sugar level in diabetic rats and increases the glucose tolerance.

Diabetes mellitus is a metabolic disorder characterized by disturbances in carbohydrate, protein, and lipid metabolism and by complications like retinopathy, microangiopathy, and nephropathy.[1] Currently available synthetic antidiabetic agents produce serious side effects like hypoglycemic coma[2] and hepatorenal disturbances.[3] Moreover they are not safe for use during pregnancy.[4] Hence, the search for safer and more effective hypoglycemic agents has continued. Following the WHO's recommendation for research on the beneficial uses of medicinal plants in the treatment of diabetes mellitus,[5] investigations on hypoglycemic agents derived from medicinal plants have also gained momentum. Several investigations have been conducted and many plants have shown a positive activity.[6] Though the active principles have been isolated from some plants, some still remain to be identified.

*Ichnocarpus frutescens* (Apocynaceae), commonly known as black creeper and ‘*dudhilata*’ is found all over India. The roots are used as a diuretic and diaphoretic.[7] The stalks and leaves are used in the treatment of skin eruptions and fever.[8] The tribals of Madhya Pradesh[9] use the roots and the siddis of Uttara Kannada district of Karnataka[10] use the roots and flowers as a cure for diabetes. So far, however, there is no scientific evidence to support the antidiabetic effect of *Ichnocarpus frutescens* (IF). The objective of this study was to ascertain the scientific basis for the use of this plant in the management of diabetes using streptozotocin-nicotinamide induced type-II diabetic rats.

## Materials and Methods

### Collection of plant material

The roots of IF were collected from the local market in Madhya Pradesh and identified by Dr. S.C. Jena, Director, Chattisgarh Rajya Van Vikas Nigam Limited, Madhya Pradesh, and Mr. A. K. Murthy, Medicinal Plants Survey, Central Ayurvedic Research Institute, Bhubaneswar, Orissa.

### Preparation of aqueous root extract of IF

The aqueous extract was prepared by cold maceration of 150 g of the shade-dried roots' powder in 500 ml of drinking water for 7 days. The extract was filtered, concentrated, dried *in vacuo* (yield 65 g), and the residue stored in a refrigerator at 2-8°C for use in the experiments.[11]

### Animals

Healthy adult male Wistar albino rats between 2-3 months of age and weighing 250-280 g were used for the study. Housed individually in polypropylene cages, maintained under standard conditions (12-h light and 12-h dark cycle; 25 ± 5°C; 35-60% humidity), the animals were fed with standard rat pellet diet (Hindustan Lever Ltd, Mumbai, India) and provided water *ad libitum*.

### Acute toxicity study

Animals were starved overnight and divided into five groups (*n* = 6). They were fed orally with the aqueous extract of IF in increasing dose levels of 100, 500, 1000, 3000, and 5000 mg/kg body weight. The animals were observed continuously for 2 h for the following:[12]

- Behavioral profile: alertness, restlessness, irritability, and fearfulness
- Neurological profile: spontaneous activity, reactivity, touches response, pain response, and gait
- Autonomic profile: defecation and urination

The number of deaths, if any were recorded after 24 and 72 h.

### Induction of non-insulin dependent diabetes mellitus (NIDDM)

NIDDM was induced[13] by a single intraperitoneal injection of 60 mg/kg streptozotocin (Sigma Aldrich, Germany) followed by nicotinamide (Ranbaxy Chemicals Ltd, Mumbai, India) 120 mg/kg, i.p, 15 min afterwards. Streptozotocin (STZ) was dissolved in citrate buffer (pH 4.5) and nicotinamide was dissolved in normal saline.[14] Hyperglycemia was confirmed by the elevated glucose levels in plasma, determined at 72 h and then on day 7 after injection. The threshold value of fasting plasma glucose to diagnose diabetes was taken as >126 mg/dl.[15] Only those rats that were found to have permanent NIDDM were used for the study.

### Collection of blood and determination of serum glucose

Blood was withdrawn from the retroorbital sinus under ether inhalation anesthesia and glucose levels were estimated using a glucose oxidase-peroxidase reactive strips and a glucometer (Accu-Chek, Roche Diagnostics, USA).

### Oral glucose tolerance test (OGTT)

The oral glucose tolerance test[16] was performed in overnight (18-h) fasted normal rats. Rats divided into four groups (*n* = 6) were administered either drinking water or IF aqueous extract, 250 and 500 mg/kg, respectively. Glucose (2 g/kg) was fed 30 min after the administration of the extract. Glibenclamide (10 mg/kg) was used as the standard drug. Blood was withdrawn from the retroorbital sinus under ether inhalation anesthesia at 30, 60, and 120 min of glucose administration and glucose levels were estimated using glucose oxidase-peroxidase reactive strips and a glucometer (Accu-Chek, Roche Diagnostics, USA).

### Experimental design for antidiabetic study

Overnight fasted diabetic rats were divided for the antidiabetic study in the following manner: Group I: diabetic control rats administered gum acacia daily for 15 days; Group II: diabetic rats administered IF aqueous extract (250 mg/kg); Group III: diabetic rats administered IF aqueous extract (500 mg/kg); Group IV: diabetic rats administered the standard drug glibenclamide (0.25 mg/kg) for 15 days.

The fasting blood glucose levels were determined on day 0, 10, and 15. During the experimental period, the rats were weighed daily and the mean change in body weight was calculated.

### Statistical analysis

Data were statistically evaluated using one-way ANOVA, followed by *post hoc* Sheffe's test using SPSS, version 7.5. The values were considered significant when *P* < 0.05.[17]

## Results

### Effect of IF aqueous root extract on acute toxicity study

Acute toxicity studies revealed the nontoxic nature of the aqueous root extract of IF. There were no lethality or toxic reactions found at any of the doses selected until the end of the study period. All the animals were alive, healthy, and active during the observation period.

### Effect of IF aqueous root extract on oral glucose tolerance test

In OGTT, the aqueous extract induced significant reduction in plasma glucose levels from 30 min onwards [Table 1].

**Table 1:** Effect of ***Ichnocarpus frutescens*** (IF) on oral glucose tolerance test in normal rats

*Group*	*Treatment*	*Plasma glucose concentration* (mg/dl)		
		
		*30 min*	*60 min*	*120 min*
1	Control glucose	(2 g/kg) 110.00 ± 1.31	101.53 ± 2.54	88.62 ± 2.55
2	IF (250 mg/kg)	66.48 ± 0.54^a^	89.41 ± 2.33	83.54 ± 1.36^a^
3	IF (500 mg/kg)	72.00 ± 1.73^a^	90.54 ± 1.44^a^	72.24 ± 1.52^a^
4	Glibenclamide (10 mg/kg)	82.34 ± 0.18^a^	76.12 ± 0.12^a^	70.45 ± 0.14^a^

Values are mean ± SD; *n* = 6 in each group

a *P* < 0.05 when compared with normal control

### Effect of IF aqueous root extract on fasting blood sugar level

On repeated administration of the extract for 15 days, a significant decrease in the fasting blood sugar level was observed in the diabetic rats as compared to the control [Table 2].

**Table 2:** Effect of IF on fasting blood sugar level in noninsulin-dependent diabetic rats

*Group*	*Treatment*	*Fasting blood sugar level (mg/dl)*		
		
		*0 day*	*10^th^ day*	*15^th^ day*
1	Vehicle	210.35 ± 1.41	220.56 ± 2.35	242.56 ± 2.53
2	IF (250 mg/kg)	205.05 ± 1.45	166.23 ± 1.81^a^	143.23 ± 2.13^a^
3	IF (500 mg/kg)	200.38 ± 4.84	152.44 ± 1.56^a^	109.13 ± 2.42^a^
4.	Glibenclamide (0.25 mg/kg)	200.74 ± 8.41	123.43 ± 3.21^a^	102.34 ± 1.36^a^

Values are mean ± SD; *n*= 6 in each group

a *P* < 0.05 when compared with diabetic control

### Effect of IF aqueous root extract on changes in body weight

Diabetic rats showed a decrease in body weight during the experimental period; this was significantly antagonized by the extract [Table 3].

**Table 3:** Effect of IF on body weight in noninsulin-dependent diabetic rats

*Group*	*Treatment*	*Body weight changes (g)*		
		
		*0 day*	*10^th^ day*	*15^th^ day*
1	Vehicle	235.12 ± 1.42	223.14 ± 2.11	210.25 ± 1.64
2	IF (250 mg/kg)	212.22 ± 1.21	200.32 ± 0.51	207.41 ± 1.12
3	IF (500 mg/kg)	224.10 ± 2.14	210.43 ± 0.18	220.35 ± 0.44^a^
4	Glibenclamide (0.25 mg/kg)	225.12 ± 1.31	218.32 ± 1.45^a^	224.35 ± 0.65^a^

Values are mean ± SD; *n*= 6 in each group.

a *P* < 0.05 when compared with diabetic control

## Discussion

The present work has detected the antidiabetic effect of the aqueous roots extract of *Ichnocarpus frutescens* in streptozotocin-nicotinamide induced type-II diabetic rats. Streptozotocin-nicotinamide injection caused diabetes mellitus, probably due to destruction of the β-cells of the islets of Langerhans of the pancreas.[18] Over-production of glucose and decreased utilization by the tissues form the fundamental basis of hyperglycemia in diabetes mellitus.[19]

When IF aqueous roots extract was administered to glucose-loaded normal rats, hypoglycemia was observed after 30 min, with the maximum effect being seen at 2 h. Our investigations also indicate the efficacy of the aqueous extract in the maintenance of blood glucose levels in normal and streptozotocin-nicotinamide induced diabetic rats. Induction of diabetes with STZ is associated with a characteristic loss of body weight, which is due to increased muscle wasting[20] and loss of tissue proteins.[21] Diabetic rats treated with the IF extract showed an increase in body weight as compared to the diabetic control, which may be due to its effect in controlling muscle wasting, i.e., by reversal of antagonizing.[22]

## Conclusion

The aqueous extract of the roots of *Ichnocarpus frutescens* has antidiabetic activity as it lowers serum glucose levels in diabetic rats and significantly increases glucose tolerance. It also increases the body weight of diabetic rats. Hence, long-term studies of *Ichnocarpus frutescens* and its isolated compounds are necessary to elucidate the exact mechanism of action so as to develop it as a potent antidiabetic drug.

## References

[1] Akhtar FM, Ali MR (1980). Study of the anti diabetic effect of a compound medicinal plant prescription in normal and diabetic rabbit. J Pak Med Assoc.

[2] Larner J, Gilman AG, Goodman LS, Rall IW, Murad F Insulin and oral hypoglycemic drug. Glucogan. The Pharmacological basis of therapeutics.

[3] Suba V, Murugesan T, Arunachalam G, Mandal SC, Sahu BP (2004). Anti diabetic potential of *Barleria lupilina* extract in rats. Phytomed.

[4] Rahman Q, Zaman K (1989). Medicinal plants with hypoglycemic activity. J Ethnopharmacol.

[5] (1980). The WHO Expert Committee on diabetes mellitus. Technical Report Series.

[6] Shirwaikar A, Rajendran K, Dinesh C (2004). Oral antidiabetic activity of *Annona squamosa* leaf alcohol extract in NIDDM rats. Pharm Biol.

[7] Nadkarni AK (2002). Ichnocarpus frutescens. The Indian Materia Medica.

[8] Yoganarasimhan SN, Togunashi VS, Keshavamurthy KR, Govindaiah H (1982). Medicobotany of Tumkur District, Karnataka. J Eco Taxon Botany.

[9] Yousuf-Shaw H, Tripathi L, Bhattacharya S (2004). Antidiabetic plants used by tribals in Madhya Pradesh. Nat Product Radiance.

[10] Bhandary MJ, Chandrashekhar KR, Kaveriappa KM (1995). Medical ethnobotany of the Siddis of Uttara Kannada district, Karnataka, India. J Ethnopharmacol.

[11] Shirwaikar A, Rajendran K, Dinesh C, Bodla R (2004). Antidiabetic activity of aqueous leaf extract of *Annona squamosa* in streptozotocin-nicotinamide type-2 diabetic rats. J Ethnopharmacol.

[12] Turner MA (1965). Screening methods in Pharmacology.

[13] Pellegrino M, Christopher B, Michelle M, Gerard R (1998). Development of a new model of type II diabetes in adult rats administered with streptozotocin and nicotinamide. Diabetes.

[14] Shirwaikar A, Rajendran K, Barik R (2006). Effect of aqueous bark extract of *Garuga pinnata* Roxb. in streptozotocin-nicotinamide induced type-II diabetes mellitus. J Ethnopharmacol.

[15] American Diabetes Association (1998). Standards of medical care for patients with diabetes mellitus. Diabetes Care.

[16] Bonner-Weir S (1988). Morphological evidence of pancreatic polarity of beta cells within islets of langerhans. Diabetes.

[17] Shirwaikar A, Rajendran K, Punitha IS (2005). Antidiabetic activity of alcoholic stem extract of *Coscinium fenestratum* in streptozotocin-nicotinamide type 2 diabetic rats. J Ethnopharmacol.

[18] Kavalali G, Tuncel H, Goksel S, Hatemi MH (2002). Hypoglycemic activity of *Urtica pilulifera* in streptozotocin-diabetic rats. J Ethnopharmacol.

[19] Chattopadhyay R (1993). Hypoglycemic effect of *Ocimum sanctum* leaf in normal and streptozotocin diabetic rats. Indian J Exp Biol.

[20] Swanston-Flat SK, Day C, Bailey CJ, Flatt PR (1990). Traditional plant treatment for diabetes: Studies in normal and streptozotocin diabetic mice. Diabetol.

[21] Chatterjea MN, Shinde R (2002). Diabetes mellitus. Text Book of Medical Biochemistry.

[22] Whitton PD, Hems DA (1975). Glycogen synthesis in perfused liver of streptozotocin diabetic rats. Biochem J.

